# Causal Interpretation of DBSCAN Algorithm: A Dynamic Modeling for Epsilon Estimation

**DOI:** 10.3390/e28040452

**Published:** 2026-04-15

**Authors:** K. Garcia-Sanchez, J.-L. Perez-Ramos, S. Ramirez-Rosales, A.-M. Herrera-Navarro, H. Jiménez-Hernández, D. Canton-Enriquez

**Affiliations:** Facultad de Informática, Universidad Autónoma de Querétaro, Av. de las Ciencias S/N, Juriquilla, Santiago de Querétaro 76230, Mexico; jorge.luis.perez@uaq.edu.mx (J.-L.P.-R.); selene.ramirez@uaq.mx (S.R.-R.); mherrera@uaq.mx (A.-M.H.-N.); hugo.jimenez@uaq.mx (H.J.-H.)

**Keywords:** structural causal model, DBSCAN, information complexity, system dynamics, causal inference, uncertainty reduction

## Abstract

DBSCAN is widely used to identify structured regions in unlabeled data, but its performance depends critically on the selection of the neighborhood parameter ε. Traditional heuristics for estimating ε often become unreliable in high-dimensional or varying-density settings because they rely heavily on local geometric criteria and may fail under smooth transitions or topological ambiguity. This work presents a three-level perspective on DBSCAN hyperparameter selection. At the algorithmic level, ε controls neighborhood connectivity and structural transitions in clustering. At the modeling level, the ordered *k*-distance signal is approximated through a surrogate dynamical estimation framework inspired by a mass–spring–damper system. At the causal level, the resulting estimator is interpreted through interventions on its internal threshold-selection mechanism. The proposed method models the variation of ε using ordinary differential equations defined on the ordered *k*-distance signal, enabling analysis of structural transitions in density organization via a surrogate dynamical representation. System identification is performed using L-BFGS-B optimization on the smoothed *k*-distance curve, while the system dynamics are solved with the fourth-order Runge–Kutta method. The resulting estimator identifies transition regions that are structurally informative for ε selection in DBSCAN. To analyze the estimator at the intervention level, Pearl’s do-calculus is used to compute the Average Causal Effect (ACE). The method was evaluated on synthetic benchmarks and on the Covtype dataset, including scenarios with multi-density overlap and dimensionality up to $R^{10}$. The resulting ACE values, +0.9352, +0.5148, and +0.9246, indicate that the proposed estimator improves intervention-based ε selection relative to the geometric baseline across the evaluated datasets. Its practical computational cost is dominated by nearest-neighbor search, behaving approximately as O(NlogN) under favorable indexing conditions and degrading toward $O(N^{2})$ in high-dimensional or weak-pruning regimes.

## 1. Introduction

In unsupervised learning, clustering algorithms aim to identify hidden structure in data by grouping samples with similar characteristics without relying on labeled information [1]. Among the most influential techniques, the Density-Based Spatial Clustering of Applications with Noise (DBSCAN) algorithm stands out for its ability to detect dense regions of arbitrary shape while separating sparse or isolated observations as noise. In this sense, DBSCAN overcomes important limitations of partition-based methods such as K-means, which assume approximately spherical clusters and are sensitive to outliers [2], as well as some hierarchical approaches whose behavior is strongly determined by the geometry induced by the data representation [3].

Despite these advantages, DBSCAN presents a persistent practical difficulty: its sensitivity to the hyperparameters ε (neighborhood radius) and minPts (minimum number of points required to define a dense neighborhood). While minPts is often selected using relatively stable practical rules, ε plays a much more delicate role because it determines when dense components emerge, merge, or disappear. As a result, small perturbations in ε may induce substantial changes in neighborhood connectivity and therefore in the final clustering structure [4,5]. This has motivated numerous attempts to automate ε selection, ranging from heuristic use of the *k*-distance plot [6] to hierarchical or adaptive strategies [7]. However, most of these approaches remain predominantly empirical and do not formally explain why a local modification in ε can trigger a global topological change in the density structure [6,8].

In this context, there are density-based extensions like HDBSCAN and OPTICS, techniques that partially address sensitivity to the ε parameter by using generative schemes or density-based structures. These allow capturing distributions with variable density without requiring a fixed global threshold. However, the added complexity makes it difficult, in certain scenarios, to explicitly understand the importance of ε in the final cluster formation and structure. Ultimately, the challenge of estimating ε within the classical DBSCAN framework remains significant from both practical and analytical viewpoints [9,10].

Traditional heuristics such as Kneedle often rely heavily on local curvature and geometric inflection criteria [11,12]. In practice, this may lead to what we refer to as *geometric myopia*: the estimator becomes highly sensitive to noise, weak transitions, and locally ambiguous changes in the ordered signal. This issue becomes more severe in higher-dimensional settings, where distance measures progressively lose discriminative power due to the concentration of measure phenomenon [13,14]. Under these conditions, spurious structural boundaries and oversegmentation may arise, making purely geometric rules less reliable for identifying an informative threshold.

These limitations motivate the search for a more structured interpretation of ε selection. In parallel, recent work has explored ways of bringing ideas from causal discovery into unsupervised learning. Historically, causal discovery methods such as the PC algorithm [15] and structural approaches based on interventions and conditional responses [16,17] were developed to identify directed dependencies among observed variables. More recently, some of these ideas have been adapted to unsupervised settings in order to obtain more structured interpretations of variability and organization in complex data environments [18]. Rather than treating threshold selection as a purely geometric problem, this perspective opens the possibility of analyzing it as an organized estimation mechanism with explicitly defined internal dependencies.

In this work, the problem of selecting ε in DBSCAN is articulated through three distinct but related levels. First, at the algorithmic level, ε controls neighborhood formation and, therefore, affects connectivity and the global clustering structure. Second, at the modeling level, this structural sensitivity is represented through a surrogate dynamical estimator defined over the ordered *k*-distance signal. Third, at the causal level, the resulting estimator is interpreted through interventions on its internal threshold-selection mechanism. These three levels are related, but they are not equivalent and should not be interpreted as automatic consequences of one another.

Under this view, small perturbations in ε induce observable changes in neighborhood connectivity and cluster configuration at the algorithmic level. These changes motivate the introduction of a surrogate dynamical layer, but do not by themselves imply a causal interpretation. To formalize the modeling level of the proposal, a mass–spring–damper-inspired formulation is introduced over the ordered *k*-distance curve, in which derivatives and state transitions characterize structural changes in density. The resulting estimator is then analyzed through interventions on its internal modules, so that the causal reading applies to the threshold-selection mechanism rather than to the physical data-generating process itself.

Thus, the present work goes beyond a traditional geometric heuristic by articulating an algorithmic description of how ε modifies connectivity, a surrogate dynamical model for the ordered *k*-distance signal, and an intervention-based interpretation of the resulting estimator. The aim is to improve the precision of ε selection while distinguishing clearly between the algorithmic, modeling, and causal roles of the proposal.

The main contributions of this work can be summarized as follows:

- A three-level formulation of the ε-selection problem in DBSCAN, distinguishing explicitly between the algorithmic role of neighborhood connectivity, the surrogate dynamical modeling of the ordered *k*-distance signal, and the intervention-based interpretation of the resulting estimator.
- A mass–spring–damper-inspired dynamical estimator defined over the ordered *k*-distance curve, designed to identify structurally relevant transition regions for ε selection beyond purely geometric elbow heuristics.
- A Structural Causal Model (SCM)-based interpretation of the internal estimation mechanism, in which do(·) and ACE are applied at the level of the threshold-selection process rather than at the level of the physical data-generating process.
- An empirical evaluation on synthetic and real-world datasets, including clustering-quality comparisons with the Kneedle baseline, an ablation analysis of the dynamic layer, and a practical runtime characterization of the proposed pipeline.

The proposal is evaluated in three scenarios: two synthetic datasets with between 1300 and 5000 samples, and one real-world multivariate dataset with 581,013 samples. The synthetic datasets were designed to represent varying-density distributions and severe topological overlaps, where traditional hyperparameter selection often fails to isolate meaningful structures. The real-world dataset evaluates the method in a multivariate setting of dimension $R^{10}$. For each scenario, the proposed estimator is compared against the Kneedle heuristic using clustering-quality indices including Davies–Bouldin, Silhouette, and Calinski–Harabasz.

Hierarchical approaches such as HDBSCAN mitigate the varying-density problem by avoiding a single global ε threshold [19]. However, the objective of this work is not to replace the density-thresholding paradigm but to provide an interpretable mechanism for estimating ε within standard DBSCAN. For this reason, the most appropriate baseline is not HDBSCAN, but the geometric heuristic commonly used for the same estimation task, namely Kneedle.

The remainder of the article is organized as follows. Section 2 presents the theoretical foundations of the proposal. Section 3 describes the methodology. Section 4 reports the experimental results and discussion. Finally, Section 5 presents the conclusions and future work.

## 2. Theoretical Foundations

### 2.1. DBSCAN and the Role of the ε Parameter

The DBSCAN (Density-Based Spatial Clustering of Applications with Noise) algorithm separates low-density regions from high-density regions in order to identify clusters and noise [20]. This process is controlled by two parameters: ε, the neighborhood radius that defines the local adjacency structure, and minPts, which specifies the minimum neighborhood cardinality required for a point to be considered part of a dense core. While minPts is often chosen using simple practical rules, ε plays a more delicate role because it determines when dense components appear, merge, or vanish.

At the algorithmic level, ε controls neighborhood formation and therefore directly affects local connectivity in DBSCAN. Since cluster expansion is based on density reachability, even small perturbations in ε may alter adjacency relations between points and induce structural changes in the final partition. In this sense, ε acts first as the control variable of the clustering mechanism itself, before any modeling or intervention-based interpretation is introduced.

This algorithmic sensitivity motivates the modeling step adopted in the present work. Rather than treating the ordered *k*-distance curve solely as a geometric heuristic, the proposal approximates it with a surrogate dynamical layer to identify structurally relevant transitions. At this stage, however, the role of the dynamic formulation is representational: it provides a structured approximation of the ordered signal and should not yet be interpreted as a causal claim about the data’s physical origin.

Although DBSCAN presents inherent limitations due to its reliance on a global density threshold, the proposed dynamical framework does not eliminate these constraints. Instead, it provides a structured way to analyze and mitigate their effects through a transition-sensitive interpretation of ε.

For this dynamic formulation to be informative, the data must exhibit specific topological properties. The framework does not require symmetry or adherence to a strict parametric distribution, but it does require the following conditions:

- The transitions between dense cluster cores and sparse ambient noise cannot be entirely discrete. The density must decay at a measurable rate so that the *k*-distance graph remains smooth and continuously differentiable.
- Data points must form continuous local manifolds. In scenarios with purely uniform, completely random noise, the dynamic layer lacks a structural anchor, preventing the system from identifying a meaningful equilibrium.
- Although the framework is designed to handle varying densities, the dataset must contain discernible density drops where cohesive structures separate from the background noise. These transitions provide the structural contrast required for the dynamical analogy to be informative.

Formally, the model aims to locate the critical transition region in the *k*-distance curve, that is, the point at which a small local variation in ε is associated with a global structural change in density connectivity. This transition provides the basis for selecting an ε value that is structurally informative for clustering.

### 2.2. *K*-Distances Curve Extraction

The *K*-distance curve characterizes the local density distribution of the data points by quantifying the distance from each data point to its *k*-th nearest neighbor [8]. In this context, the *K*-distance plot is obtained by calculating the *k* nearest neighbors of each point using Euclidean distance and the Nearest Neighbors algorithm.

First, Euclidean distance is defined as a metric that estimates the distance between two points within a system in $R^{2}$ [21], represented in Equation (1):

(1) $$d\left(x,y\right)=\sqrt{\sum_{i=1}^{d}{\left(x_{i}-y_{i}\right)}^{2}}$$

Secondly, the Nearest Neighbors algorithm can be defined as follows: given a finite dataset *S* in a Euclidean space $E^{d}$ and a query *q*, Nearest Neighbors obtains the *k* nearest neighbors *R* of *q* by evaluating δ(x,q) where x∈S [22]. The parameter *R* is defined by Equation (2).

(2) $$R=\underset{R\subset S,|R|=k_{\min}}{\arg\min}\sum_{x\in R}\delta \left(x,q\right)$$

Analogously, in clustering, neighborhoods define which points causally influence the local density and the subsequent formation of clusters.

### 2.3. Curve Smoothing

One of the most important prerequisites for working with signals is a clean input signal. When working with a *k*-distance curve, the signal is implicitly noisy, which makes numerical analysis difficult. In this study, a Savitzky–Golay filter was employed. This digital smoothing filter is based on least-squares polynomial approximation and is widely used to reduce noise in data while effectively preserving the signal’s essential characteristics, such as peak heights and widths [23]; its mathematical expression is represented by Equation (3)

(3) $$y_{i}^{\left(m\right)}=\frac{m!}{\Delta t^{m}}\sum_{j=-n}^{n}c_{j}^{\left(m\right)}\;x_{i+j}$$

where $y_{i}^{\left(m\right)}$ is the *m*-th smoothed derivative at point *i*, $x_{i+j}$ are the signal values, $c_{j}^{\left(m\right)}$ are the Savitzky–Golay filter coefficients obtained by fitting a polynomial of degree *p* over a window of 2n+1 points, Δt is the time step of the samples, and *m* is the order of the derivative to be calculated. To ensure reproducibility and algorithmic adaptability across varying dataset sizes, the filters were systematically constrained rather than manually fine-tuned. First, a cubic polynomial was selected because it is the lowest-order polynomial capable of modeling inflection points; this allows the algorithm to accurately capture structural transitions in the density space without introducing Runge’s Phenomenon (overfitting oscillations). Second, instead of a static value, the window size is defined dynamically as a proportion of the total dataset size *N*, ensuring that the resulting integer is odd and that the baseline is at least 5 points. This adaptive, scale-invariant windowing guarantees a receptive field broad enough to capture macro-structural density drops while mitigating micro-noise.

### 2.4. Mass–Spring–Damper System

A mass–spring–damper system is a dynamic system consisting of a mass *m* attached to a spring with a stiffness constant *k* and a damper with coefficient *b*, subjected to a force *F* [24]. First, the stiffness constant *k* is a magnitude that quantifies how rigid a spring is; it relates the force applied to the spring with the displacement produced by it, following Hooke’s Law [25]. This law is detailed in Equation (4)

**Figure 1:**
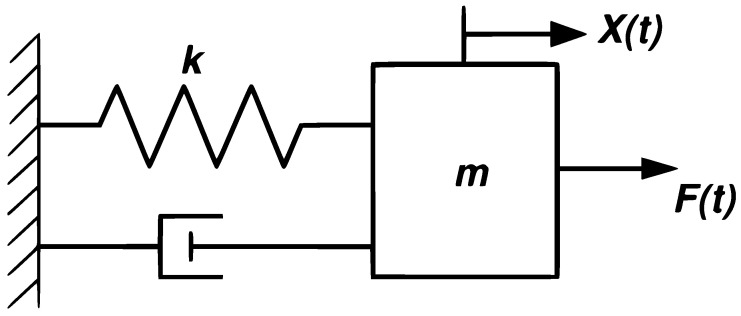
Representation of a mass–spring–damper dynamic system [27].

(4) F=kx

where *F* is the force exerted, *k* is the stiffness constant whose unit is N/m, and *x* is the displacement generated by the spring. Secondly, the damping coefficient *b* quantifies a system’s ability to dissipate kinetic energy through frictional forces [26]; its value depends on the nature of the restrictive medium. Figure 1 depicts a classic model of the mass–spring–damper system; in this representation, the interaction between the mass (*m*), the spring stiffness constant (*k*), the damping coefficient (X(t)), and the applied force (*F*) is illustrated.

Mathematically, the dynamics of the system are described by the second-order differential equation represented in Equation (5).

(5) $$m\ddot{x}\left(t\right)+b\dot{x}\left(t\right)+kx\left(t\right)=F\left(t\right)$$

At the modeling level, F(t) is introduced as the control input of the surrogate dynamic layer. Its causal interpretation is addressed later at the level of the internal estimation mechanism. That is, a change in ε leads to a perceptible alteration in the system’s acceleration and, consequently, in the cluster configuration. To solve the differential equation, the fourth-order Runge–Kutta method was used. It is a numerical method for solving differential equations. It attempts to find a numerical solution by approximating values $x_{j}$ at a finite set of points $t_{j}=t_{0}+jh$, with j={1,2,…,m} [28]. The discrete numerical integration process is expressed in Equation (6):

(6) $$\begin{array}{cc} k_{1} & =h\;f(t_{n},x_{n}),\\ k_{2} & =h\;f\left(t_{n}+\frac{h}{2},\;x_{n}+\frac{k_{1}}{2}\right),\\ k_{3} & =h\;f\left(t_{n}+\frac{h}{2},\;x_{n}+\frac{k_{2}}{2}\right),\\ k_{4} & =h\;f\left(t_{n}+h,\;x_{n}+k_{3}\right),\\ x_{n+1} & =x_{n}+\frac{1}{6}\left(k_{1}+2k_{2}+2k_{3}+k_{4}\right) \end{array}$$

This numerical integration formalizes the mass–spring–damper-inspired layer as a discrete state-space model for the ordered signal. At this point, the formulation is modeling-oriented: it defines how the surrogate dynamic state evolves before any intervention-based interpretation is introduced. Consequently, manipulating the density threshold produces an observable and mathematically traceable effect within the surrogate dynamic layer, making the internal decision mechanism explicit. It’s important to mention that the system parameters (*m*,*b*,*k*) are not treated as new manual hyperparameters; instead, they are autonomously estimated from the ordered *k*-distance representation through the fitting procedure via system identification algorithms, ensuring that the model remains objective and fully independent of user intervention.

### 2.5. System Parameter Identification

The proposed algorithm is framed within the context of system identification, a field dedicated to constructing mathematical models of dynamic systems from experimental input-output data and stochastic disturbances [29]. In this project, the identification process follows a series of structured steps to estimate the dynamic-layer parameters that constitute the system, which arise directly from the data.

Because DBSCAN operates on a finite collection of elements, the continuous theoretical model must be bridged with the dataset’s discrete nature. The *k*-distance curve is treated as a discrete ordered signal x(i), where the independent variable is an index over sorted points rather than physical time. From this ordered signal, we compute first- and second-order numerical derivatives (but not limited), which are used as descriptors of local structural variation along the sorted *k*-distance curve.

In terms of implementation, the continuous derivatives—velocity $\dot{x}\left(t\right)$ and acceleration $\ddot{x}\left(t\right)$ are numerically approximated using central finite differences over the smoothed *k*-distance array. The discrete velocity $v_{i}$ and acceleration $a_{i}$ at index *i* are computed following Equation (7).

(7) $$v_{i}\approx \frac{x_{i+1}-x_{i-1}}{2},\;a_{i}\approx \frac{v_{i+1}-v_{i-1}}{2}$$

These discrete arrays represent the observed dynamics of the data density, formally denoted as $x_{obs}$, ${\dot{x}}_{obs}$, and ${\ddot{x}}_{obs}$, moving forward. On the other hand, the objective of system identification is to find the optimal values of *m* (mass), *b* (damping), and *k* (stiffness) described in Equation (5). To achieve this, a least-squares optimization is performed; by rearranging Equation (5), the acceleration (the term to be predicted) can be isolated (see Equation (8)).

(8) $$\hat{\ddot{x}}\left(t\right)=\frac{1}{m}\left[F\left(t\right)-b\dot{x}\left(t\right)-kx\left(t\right)\right]$$

The objective function to be minimized is the mean squared error between the observed acceleration (${\ddot{x}}_{obs}\left(t\right)$) and the predicted acceleration ${\ddot{x}}_{pred}\left(t\right)$. The process is summarized in Equation (9):

(9) $$f\left(\theta \right)=\sum {\left({\ddot{x}}_{obs}\left(t\right)-{\ddot{x}}_{pred}\left(t,\theta \right)\right)}^{2}$$

For the force F(t), we assume a simple Newtonian force F(t)=C that acts as an initial excitation to set the system in motion.

Finally, a parameter estimation is performed to find the parameter vector θ=[m,b,k]. This vector is detailed in Equation (10)

(10) $$\vec{\theta }=\underset{\theta }{\arg\min}f\left(\theta \right)$$

More specifically, the cost function f(θ) is understood as a function R → R that measures how costly a set of parameters is. To perform this type of optimization, a quasi-Newtonian method called Broyden–Fletcher–Goldfarb–Shanno (BFGS) was used. This method attempts to update an approximation to the inverse Hessian matrix. This update follows Equation (11):

(11) $$H_{k+1}=H_{k}+\frac{y_{k}y_{k}^{T}}{y_{k}^{T}s_{k}}-\frac{H_{k}s_{k}s_{k}^{T}H_{k}}{s_{k}^{T}H_{k}s_{k}}$$

where $s_{k}$ is $\theta_{k+1}-\theta_{k}$, and $y_{k}$ is $\nabla f\left(\theta_{k+1}\right)-\nabla f\left(\theta_{k}\right)$. On the other hand, the update step is described in Equation (12):

(12) $$\theta_{k+1}=\theta_{k}-\alpha_{k}H_{k}^{-1}\nabla f\left(\theta_{k}\right)$$

In this way, we understand that the parameters *m*, *b*, and *k* are estimated, and that the model itself selects them based on the *k*-distance curve; this assertion is corroborated in Section 4.1.

### 2.6. Elbow Point Estimation

The elbow point of a function f(x) is a random and spontaneous event on the curve that marks the end of one state and the beginning of another [6]. In DBSCAN, the elbow point represents the change from a dense region to a sparse region (noise); the value of this point on the *y*-axis represents the value of the neighborhood radius ε.

In the proposed system, assuming a particle moves along the *k*-distance curve under the action of stiffness and damping forces, the calculation of the jerk of the particle in each iteration is of vital importance, whose formula is represented in Equation (13):

(13) $$J\left(t\right)=\frac{d\ddot{x}\left(t\right)}{dt}$$

The location where J(t) reaches its local maximum indicates a sharp change in the system’s acceleration, which in turn indicates the transition between dense and sparse areas. The ε value related to this point is therefore considered optimal.

### 2.7. Structural Causal Model

To avoid conflating distinct levels of interpretation, the proposed framework should be read in three layers. First, at the algorithmic level, ε controls neighborhood formation and, in turn, affects the connectivity and global clustering structure of DBSCAN. Second, at the modeling level, the ordered *k*-distance signal is approximated using surrogate mass–spring–damper-inspired dynamics to identify structurally relevant transitions. Third, at the causal level, the resulting estimator is interpreted through interventions on its internal modules, so that do(·) and ACE are understood as acting on the threshold-selection mechanism rather than on the physical data-generating process. These three levels are related, but they are not equivalent and should not be interpreted as automatic consequences of one another.

At the causal level, the proposed estimator is interpreted through interventions on its internal threshold-selection mechanism. In this context, do(M=·) does not denote an intervention on the physical data-generating process, but rather on the computational mechanism used to estimate ε. Under this reading, the role of the SCM is to organize the estimator’s internal dependencies and to provide an intervention-based interpretation of how changes to the threshold-selection mechanism affect clustering quality.

This section presents the SCM used to formalize the parameter estimation process, integrating directions of dependency, functional mechanisms, and controlled interventions in the sense of Pearl’s causal theory [16]. Unlike standard causal discovery, which seeks physical relationships among observed variables, the present approach models the algorithmic pipeline itself as a causal system. Under this framework, the computational steps are treated as modular mechanisms that propagate information toward the final estimate of ε.

To compare the proposed method against standard heuristics, we introduce the do-operator applied to mechanism selection. In this setting, *M* denotes the internal mechanism used to estimate ε:

- do(M=Kneedle): forces the system to select ε based on geometric curvature.
- do(M=Dynamic): forces the system to select ε based on the proposed jerk-sensitive dynamic estimator.

Conceptually, this operation corresponds to an algorithmic intervention in which the threshold-selection mechanism is replaced while the rest of the pipeline is kept fixed. This allows us to evaluate the effect of the estimation mechanism on the final clustering quality *Q*.

The total process unfolds through a set of internal variables organized hierarchically:

- *U*: exogenous variables associated with intrinsic variability and measurement noise.
- $X_{knn}$: k-NN distances and local density features after standardization.
- $S_{sg}$: smoothed signal obtained through the Savitzky–Golay filter.
- $D_{rk4}$: dynamic trajectory generated from the equation of motion and integrated using the fourth-order Runge–Kutta method.
- *J*: jerk, obtained as the third numerical derivative of the trajectory.
- $\varepsilon^{*}$: final value of the ε parameter used in DBSCAN.

**Figure 2:**
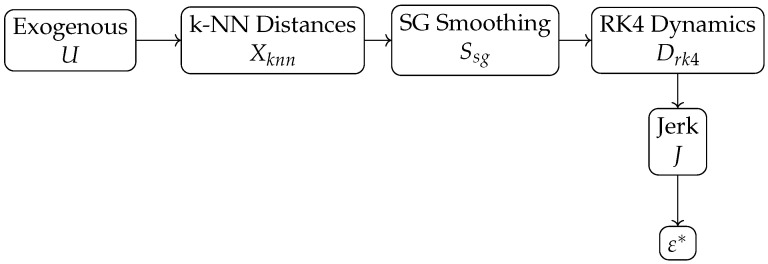
Internal causal structure of the SCM for the estimation of ε.

These variables define a unidirectional dependency structure that can be represented by the DAG shown in Figure 2. The dataset affects the smoothed signal, the smoothed signal induces the dynamic trajectory, the trajectory determines the internal transition descriptor, and this descriptor is used to produce the final estimate of ε.

The causal relationships specified in the DAG are formalized through the structural equations in Figure 2: … w given by $\varepsilon^{*}=f_{5}\left(J\right)$, so that interventions on internal nodes induce the surrogate dynamic layer. The SCM formulation assumes that each node depends uniquely on its causal parents, preserving the factorization imposed by the DAG.

After defining the structural equations, controlled interventions can be expressed using Pearl’s do(·) notation, in which the structural equation for a node is replaced externally to study how the change propagates to the output variable. In this proposal, the parameter selection is expressed as $\varepsilon^{*}=f_{5}\left(J\right)$ so that interventions on internal nodes generate observable changes in the final estimate. Formally, these interventions take the form $do(J=j_{0})$, $do(S_{sg}=s_{0})$, or $do(\theta =\theta_{0})$, leading to interventional distributions such as $P(\varepsilon^{*}|do\left(J=j_{0}\right))$.

Finally, the model’s internal coherence is examined through controlled interventions that probe the consistency of the dependency sequence. The manipulations considered are: (i) adjusting the smoothing window through $do(h=h_{0})$; (ii) modifying the dynamic parameters through $do(\theta =\theta_{0})$; and (iii) imposing artificial trajectories through $do(D_{rk4}=d_{0})$. In each case, the following dependency chain is expected:

$$\Delta S_{sg}\Rightarrow \Delta D_{rk4}\Rightarrow \Delta J\Rightarrow \Delta \varepsilon^{*}$$

This behavior is consistent with the interpretation that the parameter estimate is produced by a modular intervention-sensitive mechanism within the estimator, rather than by isolated local correlations alone.

### 2.8. Evaluation Metrics

To provide an objective description of the quality of the generated clusters, three metric categories were employed. In the first place, it is evaluated whether the data tends to cluster. For this purpose, the Hopkins statistic is used as a quantitative tool to determine whether a dataset exhibits a naturally clusterable structure [30]. In this context, the hypothesis shown in Equation (14) is applied:

(14) $$\left\{\begin{array}{c} H_{0}:H=0.5\;\left(\mathrm{Random}\;\mathrm{Distributed}\;\mathrm{Data}\right)\\ H_{1}:H<0.5\;\left(\mathrm{Tend}\;\mathrm{to}\;\mathrm{be}\;\mathrm{clustered}\;\mathrm{Data}\right) \end{array}\right.$$

In this context, the value of the Hopkins statistic is obtained from Equation (15):

(15) $$H=\frac{\sum_{i=1}^{n}\omega_{i}}{\sum_{i=1}^{n}u_{i}+\sum_{i=1}^{n}\omega_{i}}$$

where $\omega_{i}$ are the *n* sampled real points and $u_{i}$ are the *n* random points.

Next, cohesion metrics are evaluated; these assess the internal compactness of the formed clusters. In this context, we speak of inter-cluster variance ($var(C_{k})$), cluster diameter ($Diam(C_{k})$), and mean cluster distance ($Dist(C_{k})$). These metrics are described in Equations (16)–(18) respectively:

(16) $$\begin{array}{cc} \mathrm{Var}(C_{k}) & =\sum_{p\in C_{k}}{∥p-\mu_{k}∥}^{2} \end{array}$$

(17) $$\begin{array}{cc} \mathrm{Diam}(C_{k}) & =\max_{p_{i},p_{j}\in C_{k}}∥p_{i}-p_{j}∥ \end{array}$$

(18) $$\begin{array}{cc} \mathrm{Dist}(C_{k}) & =\frac{1}{|C_{k}\left|(\right|C_{k}\left|-1\right)}\sum_{p_{i}\neq p_{j}\in C_{k}}∥p_{i}-p_{j}∥ \end{array}$$

Note that when more than one cluster is found, these metrics are averaged across all *K* clusters.

Finally, separation metrics or internal validation indices were evaluated to assess the quality of the cluster partition by measuring how well the clusters are separated from one another. In this context, we find the Silhouette coefficient (measures how similar a point *i* is to its cluster; this metric is known to favor convex clusters), the Davies–Bouldin index (measures the ratio of the worst similarity between clusters), and the Calinski–Harabasz index or variance ratio (measures the relationship between the variance between clusters and the variance within the clusters). Their calculations are described in Equations (19)–(21) respectively:

(19) $$\begin{array}{cc} s(i) & =\frac{b(i)-a(i)}{\max(a(i),b(i))} \end{array}$$

(20) $$\begin{array}{cc} \mathrm{DB} & =\frac{1}{K}\sum_{k=1}^{K}\max_{l\neq k}\left(\frac{s_{k}+s_{l}}{M_{k,l}}\right) \end{array}$$

(21) $$\begin{array}{cc} \mathrm{CH} & =\frac{B_{K}\left(N-K\right)}{W_{K}\left(K-1\right)} \end{array}$$

## 3. Materials and Methods

### 3.1. Datasets

#### 3.1.1. Real-World Dataset: Covtype

The method was tested on the *covtype* dataset [31], a large-scale, real-world reference dataset derived from classifying forest cover types. The dataset contains 581,013 samples and 57 attributes. It should be noted that only the first 10 variables were used, as they are continuous numerical variables. Because it is derived from physical topography, it does not follow a simple parametric distribution; rather, it exhibits a complex, unknown multivariate distribution characterized by smooth density gradients and diffuse class boundaries. The model’s ability to identify global thresholds is tested in non-synthetic environments with significant dimensionality ($R^{10}$). The selected features are standardized to a mean of 0 and a variance of 1 before calculating neighborhood distances so that no feature dominates the Euclidean distance due to their different scales.

#### 3.1.2. Synthetic Datasets

Two synthetic benchmarks are created to emphasize the known error patterns of the ε heuristic, which are based on curvature: (i) variable density clusters that consider strong and uniform background noise, and (ii) smooth changes in density, which produce an S-curve of distance *k* and a confusing knee structure. The synthetic dataset 1 generates a mixture of homogeneous samples that simulate diffuse background interference and Gaussian spots representing dense cores. On the other hand, synthetic dataset 2 is produced by superimposing Gaussian components. They are arranged to create a continuous density gradient and deliberately reduce the number of low-density valleys, which could explain several isolated clusters. Both synthetic datasets were generated using a combination of sklearn.datasets.make_blobs and numpy.random.uniform, using a global seed of 23 (see Table 1).

### 3.2. General Scheme of the Proposal

**Figure 3:**
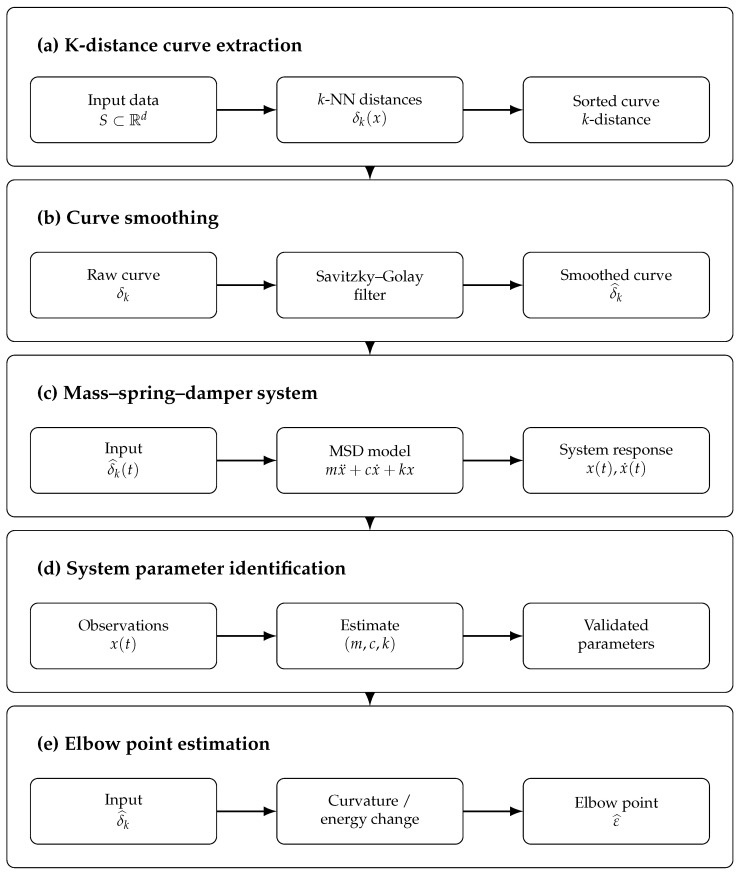
Dynamic DBSCAN algorithm block diagram.

Figure 3 summarizes the flow of the proposed explicit-causal framework. Conceptually, the *k*-distance curve extraction and smoothing processes are based on Section 2.2 and Section 2.3, and the mass–spring–damper dynamic modeling system, system parameter identification, and elbow point estimation are founded on Section 2.4, Section 2.5 and Section 2.6, respectively. This section represents the specific computational implementation of these bases.

### 3.3. Algorithm Implementation

The development of the proposed explicit-causal model faces three main challenges: the sensitivity of the ε parameter to local density variability, the mathematical discretization of continuous physical dynamics into a finite data space, and the inherent risk of replacing a single manual hyperparameter with multiple new ones. To tackle the first challenge—where analyzed datasets with dimensions up to $R^{10}$ often generate misleading peaks in the *k*-distance curve—we applied a Savitzky–Golay filter. This filter effectively reduced noise, preserving local curvature and critical inflection points, without altering the overall signal trend. To solve the second, central finite differences were used to bridge the continuous mass–spring–damper equations and the discrete nature of the sorted distance array. Finally, the third challenge was overcome by framing the formulation as a system identification problem; by utilizing L-BFGS-B optimization, the system’s dynamic-layer parameters (*m*, *b*, and *k*) are autonomously derived directly from the ordered *k*-distance representation of the data, ensuring a fully objective and user-independent model.

Finally, Algorithm 1 refers to the explicit-causal process. This algorithm takes as input parameters: *X*, a dataset of dimension N×d; *k*, the neighborhood parameter used by DBSCAN; $\theta_{0}$, the proposed dynamic-layer parameters of the dynamic system (mass, damping, and stiffness); and *h*, the time step for RK4. On the other hand, the algorithm returns the optimal values of the ε parameter and *C*, as well as the cluster labels generated by DBSCAN, using $\varepsilon_{opt}$.
**Algorithm 1** Parameter ε Estimation using Dynamic-Causal Modeling  1:**procedure **CausalEpsilonEstimation(X,k,θ,h)  2:   $X_{std}\leftarrow \;$Standardize(*X*)                 ▹ Normalize the dataset  3:   D← ComputeKNN_Distances($X_{std},k$)           ▹ Compute *k*-th distances  4:   $D_{sorted}\leftarrow$ QuickSort(*D*)             ▹ Sort distances in ascending order  5:   $D_{smooth}\leftarrow$ SavitzkyGolay($D_{sorted},window,poly$)       ▹ Smooth *k*-distance curve  6:   θ← L-BFGS-B($\theta_{0}$)        ▹ Identification of dynamic system parameters  7:   **for** each $t\in D_{smooth}$ **do**  8:         Solve ODE RK4  9:         Calculate acceleration a(t) and jerk J(t)10:   **end for**11:   $t^{*}\leftarrow \arg\max_{t}\left(J\left(t\right)\right)$               ▹ Locate global maximum of the jerk12:   $\varepsilon_{opt}\leftarrow D_{smooth}\left[t^{*}\right]$                      ▹ Determine optimal ε value13:   Labels← DBSCAN($X_{std},\varepsilon_{opt},k$)          ▹ Apply DBSCAN with optimal ε14:   **return** $\varepsilon_{opt},\mathrm{Labels}$15:**end procedure**

### 3.4. Configuration and Hyperparameters

Table 2 summarizes the hyperparameters and initial configurations established for the proposed structural causal model, the signal processing stage, and the Kneedle algorithm parameters. These values were kept constant across all datasets to ensure strict experimental reproducibility and comparability. To prevent overfitting during the smoothing phase, the Savitzky–Golay polynomial degree was strictly set to a cubic form according to Section 2.3. Furthermore, instead of a static value, the window size (*w*) was defined dynamically as approximately 5% of the dataset size *N*. For the system identification phase, the L-BFGS-B optimization was initialized with a vector $\theta_{0}$ and constrained by lower bounds to prevent invalid negative parameter values.

### 3.5. Implementation Issues

Suppose *X* is a dataset, where *N* represents the number of observations and *d* the number of variables. Let *k* be the number of minimum points (minPts), representing the number of neighbors DBSCAN uses to create the *k*-distance curve. The proposed SCM process is divided into four stages: (*i*) preparation and calculation of *k*-distances, (ii) smoothing using Savitzky–Golay, (iii) simulation of the dynamic system (RK4) with the smoothed *k*-distance signal, and (*iv*) determination of the optimal point via the maximum jerk to calculate ε. The step-by-step complexity of the process involves the following analysis:

1. Standardization and *k*-NN neighborhood: Standardization by mean and standard deviation per feature costs O(Nd). The dominant step is computing the *k*-nearest-neighbor distances. When using a precise spatial partition index, such as a *k*d tree, the estimated cost for its construction is generally O(dNlogN). However, it should be noted that in low to moderate dimensionality with favorable data distributions, the expected query time is almost logarithmic. Nevertheless, in environments with considerable dimensionality, such as in the case of the dataset with real data, pruning efficiency deteriorates due to the dimensionality. Furthermore, exact nearest neighbor search may require a linear scan per query, i.e., O(N), which implies a total cost close to $O(N^{2})$ when all points are considered. Therefore, the overall complexity of this stage is best reported as *expected* O(NlogN) in low-dimensional settings, with a *worst-case*/*high-dimensional* behavior that can degrade toward $O(N^{2})$ depending on the neighbor-search strategy and the effective dimensionality of the data. Finally, sorting the *k*-distances requires O(NlogN).
2. Smoothing using the Savitzky–Golay filter: A polynomial sliding window filter is used to process the ordered distance signal. Since the polynomial degree *p* and the window dimension *w* are fixed parameters in the implementation, the convolution procedure has a linear behavior in relation to the number of measurements, so its computational complexity is O(Nw). Nonetheless, in practice, the complexity is O(N).
3. Identified dynamics and numerical solution (RK4): In this step, each measurement of the smoothed signal is treated as a state reference within the surrogate dynamic layer. Where its temporal evolution is determined in integration intervals of size Δt. The limited-memory L-BFGS-B: method is used to optimize the dynamic system parameters, so its computational complexity is O(N). Likewise, the RK4 algorithm performs four evaluations of the acceleration function per iteration, which entails a constant cost per step; therefore, the complexity is O(N).
4. Calculation of jerk, energy, and selection of ε: The jerk is determined using finite differences, and the argmax is obtained through a linear scan. Therefore, the computational cost is O(N).

Therefore, the practical cost of the proposed pipeline is dominated by nearest-neighbor search and depends on the indexing strategy, the effective dimensionality of the data, and optimizer convergence. Under favorable spatial indexing conditions, the method behaves approximately as O(NlogN), whereas in high-dimensional or weak-pruning regimes the nearest-neighbor stage may degrade toward $O(N^{2})$. For this reason, the method should not be characterized by a single universal complexity order, but rather by a conditional computational profile determined by the geometry of the data and the search strategy employed.

## 4. Results

### 4.1. Experimental Design and Data Configuration

The experimental design aims to evaluate the proposed explicit-causal estimator, which formulates the search for the optimal DBSCAN ε parameter as a system identification problem within a mass–spring–damper-inspired framework. Specifically, the experiment evaluates how the proposed dynamical estimator responds to different topological structures in order to determine the optimal density threshold through its internal response profile.

**Figure 4:**
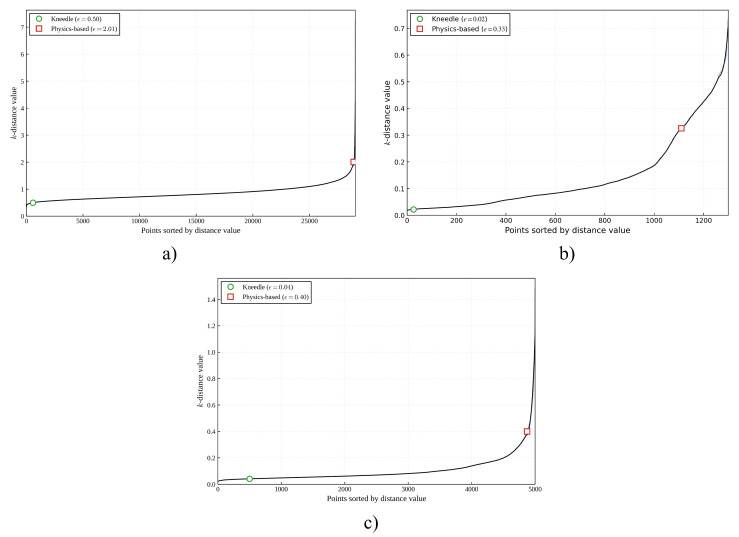
*k*-distances graphs for: (**a**) Covtype dataset, (**b**) Synthetic dataset 1, and (**c**) Synthetic dataset 2.

First, the *k*-distance graph was calculated for all datasets using Nearest Neighbors, showing that the proposed dynamical formulation identifies a candidate transition region for threshold selection. Next, Figure 4 presents these graphs for each dataset, illustrating how variations in the ordered signal are used to detect structurally relevant changes for DBSCAN.

To reinforce the algorithm’s analysis, estimates of the mass, damping, and stiffness parameters were obtained, testing the model’s adaptability across the datasets (see Table 3).

An interesting point noticed in Table 3 is the near-zero damping parameter (b≈0), which is better interpreted as an empirical property of the fitted surrogate dynamics under the current preprocessing and optimization setup. Under the present fit, low damping values suggest that the dynamic layer preserves sensitivity to abrupt structural transitions instead of attenuating them through strong dissipative terms.

In typical dynamic formulations, damping is used to suppress high-frequency oscillations induced by noise. However, in the present framework, the Savitzky–Golay filter already removes a substantial portion of the high-frequency variability while preserving the structural shape of the signal. From an estimation standpoint, this indicates that the velocity-related contribution became less influential under the fitted objective function. Therefore, the observed low-damping regime should be interpreted cautiously as a result of the current fitting configuration rather than as direct evidence of an intrinsic physical or causal property of the data.

### 4.2. Ablation Analysis of the Dynamic Layer

To assess the specific contribution of the full dynamic formulation, an ablation analysis was conducted using progressively simplified variants of the proposed method. The comparison included: (i) the full dynamic model, consisting of Savitzky–Golay smoothing, parameter fitting of the surrogate dynamic layer, RK4-based state evolution, and jerk-based ε detection; (ii) Savitzky–Golay smoothing followed by direct numerical jerk estimation without dynamic fitting; (iii) Savitzky–Golay smoothing followed by a simpler change-point detector based on the second derivative; and (iv) the Kneedle baseline. This experiment was designed to determine whether the observed clustering behavior is primarily attributable to the full structured dynamic layer or to the smoothing and transition-detection stages alone. For each variant, the estimated value of ε was used to run DBSCAN, and the resulting partitions were evaluated in terms of the number of clusters, percentage of noise, Silhouette score, Davies–Bouldin index (DBI), and Calinski–Harabasz index (CHI).

As shown in Table 4, the ablation results do not indicate a uniform advantage of the full dynamic model across all evaluated datasets. In the Covtype dataset, the variants based on Savitzky–Golay smoothing combined with direct jerk estimation or second-derivative detection produced more coherent clustering solutions than both the full model and the Kneedle baseline. A similar tendency was observed in Synthetic 1, where the simplified variants yielded substantially lower noise levels and more favorable internal clustering metrics than the full dynamic formulation. In Synthetic 2, the results were again mixed: although the full model and Kneedle produced highly fragmented solutions with large noise percentages, the simplified variants led to more compact partitions, with the second-derivative alternative achieving the most interpretable multi-cluster solution.

Overall, these results suggest that a substantial part of the practical effectiveness of the proposed pipeline is associated with the smoothing stage and the detection of structural transitions in the ordered *k*-distance signal. Under the present experimental configuration, the full surrogate dynamic layer does not consistently outperform its simplified counterparts. Therefore, the contribution of the proposed formulation should be interpreted more cautiously: not as definitive evidence that the full dynamic model is always necessary, but rather as evidence that the ordered-signal representation, together with transition-sensitive criteria such as jerk or curvature-related changes, plays a central role in ε selection.

### 4.3. Runtime and Computational Cost

To complement the asymptotic analysis, the practical computational cost of the proposed pipeline was documented using the same implementation employed in the experiments. All measurements were obtained in Google Colab under a CPU-only configuration (x86_64), with 12.7 GB of RAM and no GPU/CUDA acceleration. In all cases, nearest-neighbor retrieval was performed using the kd-tree strategy. Warm-up runs were executed before measurement, and the complete pipeline was then repeated 30 times for each dataset. Reported times correspond to mean and standard deviation in seconds.

Table 5 summarizes the observed runtime behavior. The results show that the nearest-neighbor stage dominates the total cost in the largest dataset, whereas in the synthetic datasets the optimization stage becomes more visible due to the smaller scale of the search problem. This supports the view that the practical cost of the method depends on both the neighbor-search structure and the geometry of the data.

### 4.4. Efficiency Metrics

To objectively evaluate the quality, robustness, and significance of the clusterings generated by the ε value, an empirical expectation analysis was made over 50 iterations for each dataset. For the real-world Covtype dataset, we applied a bootstrap resampling technique to capture the variance of the data distribution. For the synthetic data, the random seeds that govern the structural generation and background noise were dynamically varied. This ensures that the reported metrics represent the expected behavior of the algorithms under topological uncertainty. Two sets of metrics were applied: (i) cohesion metrics and (ii) inter-cluster separation metrics. For cohesion, we measure the Intracluster Variance (IV), the Cluster’s Mean Diameter (CMD), and the Cluster’s Mean Distance (DMD). In Table 6, we can find the results of these cohesion metrics. On the other hand, Table 7 presents the results of the inter-cluster separation metrics. The results, reported as the mean ± standard deviation across the 50 iterations, are presented in Table 6 and Table 7.

Before discussing the quality of the formed clusters, it is necessary to define the data’s clustering tendency. For this, the Hopkins statistic was used; from this context, values of 0.0773 for the Covtype dataset, 0.2646 for Synthetic 1, and 0.1027 for Synthetic 2 were obtained. The low magnitude of the statistic in all three cases indicates a tendency to cluster, consistent with the inverse formulation, where H → 0.

In this context, across the empirical expectation evaluations, Kneedle reports extremely low diameters, indicating that this method is fragmenting natural structures into very tiny components (averaging 100 clusters in Covtype, 7 clusters in Synthetic 1, and 67 in Synthetic 2), which suggests oversegmentation. On the other hand, the proposed algorithm reports clusters of greater dimension and variance; given that Hopkins ensures the data is clustered, the method’s greater variance indicates a more faithful representation of the clusters’ real extent (core and periphery).

**Figure 5:**
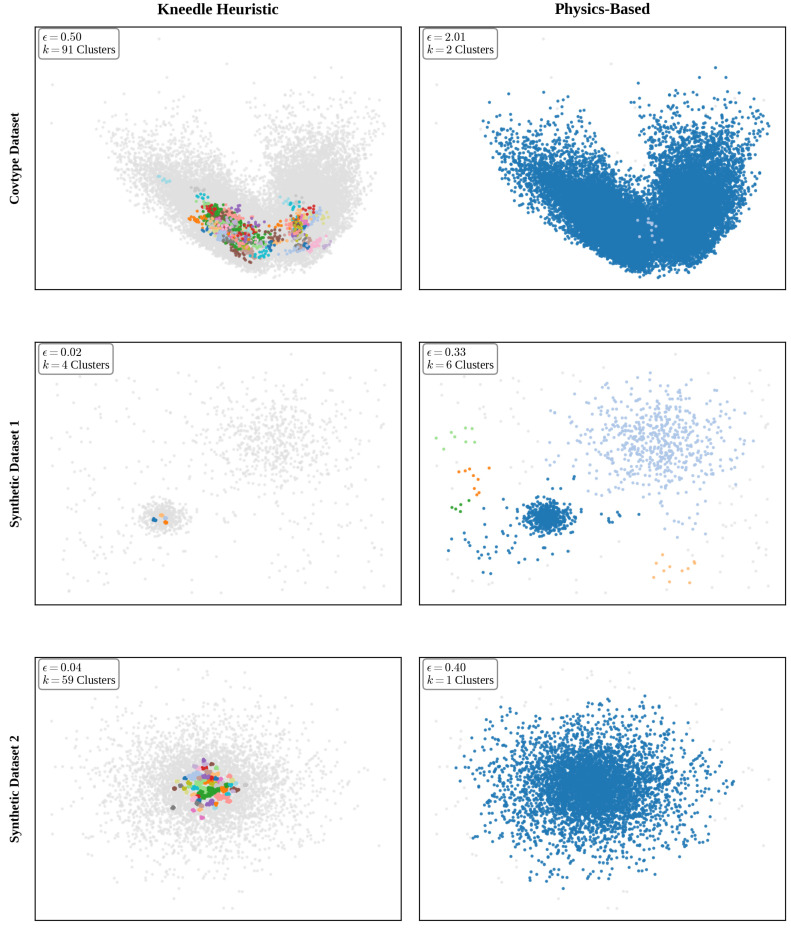
Clustering results applying both methods from a representative iteration.

These three metrics allow evaluation of the separation quality of the detected groupings; on the one hand, the Silhouette coefficient indicates that, across the three datasets presented, Kneedle consistently yields negative values, providing mathematical evidence that the partitions are artificial. In contrast, the proposed method maintains positive values (0.4202 for the Covtype dataset, 0.3132 for the Synthetic dataset 1, and 0.4588 for the Synthetic dataset 2), indicating that the data’s natural structure is preserved. This disparity highlights the Geometric Myopia of the Kneedle algorithm mentioned before. By failing to account for the dynamic inertia of the density curve, Kneedle reacts to local geometric irregularities (noise), selecting premature ratios that fracture unitary structures. Figure 5 illustrates a visual comparison of the clustering enconding each class with different colors results from a single representative iteration. The first row displays the application of both methods to the Covtype dataset, while the second and third rows show the results for the Synthetic 1 and Synthetic 2 datasets, respectively. Note that for the Covtype dataset, Principal Component Analysis (PCA) was applied to project the 10-dimensional feature space onto a 2D plane for visualization. While Table 6 and Table 7 expect over 50 runs, this visualization exemplifies the typical topological behavior previously discussed.

To quantify the benefit of the dynamic intervention over the geometric heuristic, we calculate the Average Causal Effect (ACE), understood here as the expected change in clustering quality when the ε-selection mechanism is switched from the geometric baseline to the proposed dynamic estimator (see Equation (22)). In this context, do(M=·) should be interpreted as an intervention on the internal estimation mechanism rather than on the physical data-generating process.

(22) ACE=E[Q∣do(M=Dynamic)]−E[Q∣do(M=Kneedle)]

Applying this to the experimental results:

- Covtype Dataset: As shown in Table 7, the dynamic intervention consistently corrects the massive fragmentation produced by Kneedle across 50 bootstrap samples, shifting the expected quality from negative to positive: $ACE_{covtype}=0.4202-\left(-0.5150\right)=+0.9352$
- Synthetic Dataset 1: Similarly, the intervention prevents the geometric algorithm from overfitting to uniform background noise across varying random seeds. $ACE_{syn1}=0.3132-\left(-0.2016\right)=+0.5148$
- Synthetic Dataset 2: For this dataset, Kneedle consistently fractured the structure due to ambiguous density gradients, yielding a negative expected score. The proposed dynamical decision mechanism successfully captured the dominant structural transition in the dataset: $ACE_{syn2}=0.4588-\left(-0.4658\right)=+0.9246$

### 4.5. Analysis and Causal Discussion

It is important to distinguish the proposed SCM from a physical model of the data generation process. The DAG in Figure 2 does not imply that the dataset itself was generated by a mass–spring–damper system. Instead, it assumes that the ordered *k*-distance curve can be embedded into a surrogate dynamical representation whose internal transitions help identify the decision point for ε. By intervening on the decision mechanism (do(*M*)), we show that the proposed dynamical decision mechanism isolates the intervention-sensitive cut-off point more effectively than the geometric heuristic. Thus, the causality considered here refers to the flow of information within the estimator, supporting the use of the ACE metric to quantify the algorithmic improvement produced by intervening on the threshold-selection mechanism.

However, there is a challenge in evaluating computational pipelines through a causal view; it is about distinguishing true structural causality from functional dependence. In deterministic algorithms, changing an intermediate stage would modify downstream outputs. To assess whether the proposed DAG can be interpreted as an SCM with modular intervention-sensitive components rather than a simple sequence of operations, we show that these mechanisms keep their inner structural integrity across varying input conditions and respond to targeted interventions predictably. To address this, we operationalized Pearl’s do-calculus to conduct control tests and modular forward-dependence tests across the three scenarios.

#### 4.5.1. Control Tests

To prove that the internal modules act autonomously, we performed a control test using an intervention that should not modify the topological structure or spatial orientation. Let *U* be the exogenous unobserved spatial orientation of the dataset. We intervened in the data generation mechanism by applying $do(U=U_{rot})$, where $U_{rot}$ is the 45-degree rotation matrix for the 2D synthetic datasets and a random N-dimensional orthogonal rotation matrix for the 10-dimensional Covtype dataset obtained via QR decomposition. Due to the $X_{knn}$ module, which computes isotropic distances, the downstream mechanisms should be entirely invariant to $U_{rot}$. The maximum absolute difference in the smoothed signal $S_{sg}$ before and after the intervention was recorded as follows:

- Covtype Dataset: $\Delta S_{sg}=1.77\times 10^{-15}$.
- Synthetic Dataset 1: $\Delta S_{sg}=3.69\times 10^{-16}$.
- Synthetic Dataset 2: $\Delta S_{sg}=1.33\times 10^{-15}$.

These values show a machine-level zero. This control test reflects that modules $f_{1},\ldots f_{5}$ are stable and structurally invariant to exogeneous spatial confounders, isolating the topological mechanism from coordinate variance.

#### 4.5.2. Modular Intervention

To examine whether the internal dynamic layer behaves as an intervention-sensitive component of the estimator, we applied the modular intervention do(b=100), imposing an artificial high-damping regime on the fitted response without modifying the preceding curve-extraction or jerk-computation stages. Under this intervention, the expected value of ε shifted relative to the unperturbed configuration, indicating that the estimator’s internal response is sensitive to targeted changes in the dynamic layer. The results were conclusive across all topologies:

- Covtype: Observational $E[\varepsilon^{*}]=7.2812$ vs. Intervened $E[\varepsilon^{*}|do\left(b=100\right)]=2.0096$.
- Synthetic 1: Observational $E[\varepsilon^{*}]=0.2906$ vs. Intervened $E[\varepsilon^{*}|do\left(b=100\right)]=0.2783$.
- Synthetic 2 Observational $E[\varepsilon^{*}]=0.4102$ vs. Intervened $E[\varepsilon^{*}|do\left(b=100\right)]=0.0372$.

These intervention results should be interpreted at the level of the computational mechanism rather than as evidence of a physical law in the data-generating process. In this sense, the proposed SCM provides a structured way to analyze how changes in the internal estimation modules propagate toward the final threshold selection.

#### 4.5.3. Final Remarks

Overall, the contribution of the proposed framework should be understood through three related but distinct layers: algorithmic sensitivity of DBSCAN to ε, surrogate dynamical modeling of the ordered *k*-distance signal, and an intervention-based interpretation of the resulting estimator. This separation is important because the causal reading is not presented here as an automatic consequence of the physical analogy, but as an additional interpretive layer defined over the estimator itself.

The dynamic analysis of the mass–spring–damper-inspired layer suggested that jerk peaks tend to coincide with structural transitions in the ordered density signal. This supports the use of jerk as a transition-sensitive descriptor within the proposed estimator and provides a mechanistic interpretation of the internal decision process. In contrast to Kneedle, which relies on geometric detection of the inflection point, the proposed framework uses the structured dynamical response of the signal to identify transition regions that are informative for ε selection.

Finally, the analysis of empirical expectations supports the applicability of the proposed intervention-based mechanism. In scenarios characterized by spatial overlap, with dimensionality up to $R^{10}$, background noise, and continuous density gradients, the geometric heuristic showed greater sensitivity to local irregularities. In contrast, the proposed SCM more consistently preserved the dominant structural organization of the data. The Average Causal Effect scores across the evaluated challenges are consistent with the view that intervening on the estimation mechanism can improve threshold selection relative to the geometric baseline, although the ablation analysis also indicates that an important part of this behavior is associated with the smoothing stage and the detection of structural transitions in the ordered *k*-distance signal.

### 4.6. Scope, Validity Region, and Expected Failure Cases

The proposed method should not be interpreted as a universal solution for ε estimation. Its applicability depends on whether the ordered *k*-distance signal preserves a structurally meaningful transition after smoothing. In practical terms, the method is more advisable when the boundary between dense regions and noise is smooth enough to generate a detectable transition, when the data form locally continuous manifolds, and when the resulting signal admits a stable transition-sensitive criterion such as jerk or curvature change.

Conversely, the method becomes less advisable in scenarios where the ordered *k*-distance signal does not provide a stable structural anchor. This includes cases dominated by nearly uniform noise, highly irregular signals without a coherent transition region, or geometries in which no stable and interpretable ε emerges after smoothing. Under such conditions, the dynamic layer may lose discriminative power, and the resulting threshold should be interpreted with caution.

At the same time, the method should be interpreted within a restricted validity region. Its usefulness depends on the presence of a structurally meaningful transition in the smoothed ordered *k*-distance signal, and its practical cost is determined by the nearest-neighbor search strategy and the effective dimensionality of the data. Therefore, the proposed framework is better understood as an intervention-sensitive estimator for ε selection under suitable geometric conditions, rather than as a universally transferable causal model for all density configurations.

### 4.7. Future Work

Further works that become enabled for further work that complements this proposal include the following: Developing an adaptive multi-scale strategy to enable local estimation of ε in regions with heterogeneous densities remains a promising direction for future work. Another important line of research involves extending the proposed SCM-based interpretation of DBSCAN to other clustering paradigms, including graph-based and feature-learning approaches, to broaden its applicability to non-Euclidean spaces. Further investigation could focus on combining the proposed SCM with structural inference techniques, such as the PC algorithm, to identify relationships between clustering parameters and latent factors in complex datasets. Incorporating additional internal validation measures, such as DBCV, would allow for a more comprehensive assessment of connectivity and density-based separation in non-convex cluster structures. Moreover, by contrasting the estimated partitions with the original generative structure in synthetic datasets using external validation indices such as ARI, NMI, and AMI, one can gain deeper insight into clustering performance. Expanding the comparative analysis to variable-density clustering algorithms, including HDBSCAN and OPTICS, as well as exploring ε-selection strategies beyond Kneedle and evaluating the framework on superior dimensions to $R^{10}$, including the evaluation of the modularity and invariance properties of each structural component ensuring that each function behaves as an autonomous causal mechanism under interventions in datasets, also represents a valuable extension. Finally, examining the stability of the fitted parameters (m, b, k) across multiple initializations would help determine whether the observed low-damping regime is robust or specific to certain configurations.

## 5. Conclusions

This article presents a three-level interpretation of ε selection in DBSCAN. At the algorithmic level, ε governs neighborhood connectivity and structural transitions in clustering. At the modeling level, the ordered distance signal is approximated through a structured dynamical representation inspired by a mass–spring–damper system. At the causal level, the resulting estimator is interpreted through interventions on its internal threshold-selection mechanism.

Under this view, the contribution of the paper should not be understood as an automatic passage from topological sensitivity to physical dynamics and then to causality. Instead, the proposal is organized as a layered framework in which each level plays a distinct role: algorithmic sensitivity motivates the modeling step, the modeling step provides a structured representation of the ordered signal, and the causal step offers an intervention-based interpretation of the internal estimation mechanism.

This article presents the view that the tuning of the ε hyperparameter in the DBSCAN algorithm can be interpreted as an explicit-causal estimation process supported by a structured dynamical representation of the distance distribution. By modeling the curve using differential equations, we establish a link between local variations in the slope and structural changes in data density. This reinterpretation converts the estimation of the neighborhood radius into the identification of an intervention-sensitive transition point in the ordered density signal: a slight modification in the neighborhood triggers a significant change in the cluster configuration.

The experimental results tested the method with complex scenarios known in the literature where DBSCAN fails by definition: $R^{10}$ dimensionality data (Covtype) and data with smooth density transitions (Synthetic 2). Both scenarios contribute to the development of variable-density algorithms such as OPTICS and HDBSCAN.

Despite these limitations, the empirical comparison with Kneedle shows that the proposed intervention-based estimator can produce more favorable threshold selections under the evaluated scenarios. In particular, Kneedle frequently yielded fragmented or unstable partitions, whereas the proposed framework more often preserved the dominant structural organization of the data. The ACE values support the usefulness of intervening on the estimation mechanism relative to the geometric baseline. At the same time, the ablation analysis indicates that an important part of this behavior is also associated with the smoothing stage and with the detection of structural transitions in the ordered *k*-distance signal.

This separation improves the precision of the central argument and helps delimit the scope of the claims made in the manuscript. In particular, the causal language adopted here refers to the modular computational structure of the estimator, while the dynamical layer remains a surrogate modeling device for transition detection in the ordered *k*-distance signal.

An important line of research involves extending the proposed SCM-based interpretation of DBSCAN to other clustering paradigms, including graph-based and feature-learning approaches, with the aim of broadening its applicability to non-Euclidean spaces. Afterwards this study could focus on combining the proposed SCM with structural inference techniques, such as the PC algorithm, to identify relationships between clustering parameters and latent factors in complex datasets.

Adding more internal validation measures, such as DBCV, would make it practicable to more accurately evaluate connectivity and density-based separation in non-convex cluster structures. Furthermore, comparing the estimated partitions to the original generative structure in synthetic datasets using external validation indices like ARI, NMI, and AMI would give us a better idea of how well the clustering works. Expanding the comparative analysis to variable-density clustering algorithms, including HDBSCAN and OPTICS, as well as exploring ε-selection strategies beyond Kneedle and evaluating the framework on higher-dimensional datasets, also represents a valuable extension. Finally, examining the stability of the fitted parameters (m,b,k) under multiple initializations would help determine whether the observed low-damping regime is robust or dependent on specific configurations. This comprehensive validation strategy will strengthen the generalizability of the results as intrinsic properties of the system. By extending the sensitivity of the physical parameters toward greater robustness in the clustering, an even more accurate interpretation of the damping regimes in various operating scenarios will be consolidated.

## Figures and Tables

**Table 1:** Summary of datasets used in the experiments.

Dataset	Samples (*N*)	Dim. (*d*)	Notes
Covtype	581,013	10	Continuous attributes only, standardized
Synthetic 1	1300	2	Varying density + uniform background noise
Synthetic 2	5000	2	Smooth density gradient (ambiguous knee)

**Table 2:** Configuration and hyperparameters used in the explicit-causal SCM and the comparison baseline.

Parameter	Description	Value	Usage
*k* (minPts)	Minimum points for adjacency/DBSCAN	8	*k*-NN curve extraction & Clustering
*p*	Savitzky–Golay polynomial degree	3	Curve smoothing phase
*w*	Savitzky–Golay window size	≈0.05*N* (odd, ≥5)	Adaptive curve smoothing
*F*	External Newtonian excitation force	1.0	MSD dynamic system input
$\theta_{0}$	Initial dynamic-layer parameters [m,b,k]	[1.0,1.0,1.0]	L-BFGS-B optimization initialization
Bounds	Optimization boundaries for θ	$\left[10^{-6},\infty \right)$	L-BFGS-B constraints
*h* (Δt)	Finite differences integration step	1	Discrete kinematic derivatives (RK4)
Buffer	Initial search buffer to ignore early noise	0.01N	Elbow point/Jerk estimation
$S_{kneedle}$	Sensitivity parameter for Kneedle algorithm	1.0	Baseline structural comparison

**Table 3:** Estimation of each parameter from the model.

Dataset	Mass	Stiffness	Calculated ε
Covtype	2561.3921	1.0503	2.0082
Synthetic Dataset 1	1538.9829	3.3571	0.3764
Synthetic Dataset 2	2235.5416	4.1494	0.4003

**Table 4:** Ablation analysis of the proposed dynamic layer for ε estimation.

Dataset	Method	ε	Clusters	Noise (%)	Silhouette	DBI	CHI
Covtype	Full dynamic model	7.2812	1	0	N/A	N/A	N/A
Covtype	SG + direct jerk	2.0082	2	0.14	0.4124	1.7303	68.6748
Covtype	SG + 2nd derivative	2.0096	2	0.14	0.4138	1.6889	69.9627
Covtype	Kneedle	0.4986	87	93.57	−0.0504	1.4870	11.3456
Synthetic 1	Full dynamic model	0.0212	8	94.69	−0.2098	1.1388	5.9502
Synthetic 1	SG + direct jerk	0.3026	5	10.54	0.0109	2.0348	36.7928
Synthetic 1	SG + 2nd derivative	0.3028	5	10.38	0.0151	1.999	37.7267
Synthetic 1	Kneedle	0.0223	7	93.46	−0.1924	1.1252	8.5627
Synthetic 2	Full dynamic model	0.037	53	85.98	−0.5017	3.7719	1.4211
Synthetic 2	SG + direct jerk	0.4003	1	1.26	N/A	N/A	N/A
Synthetic 2	SG + 2nd derivative	0.1972	3	7.14	0.1784	8.3023	27.0966
Synthetic 2	Kneedle	0.0416	64	77.44	−0.4655	3.2747	2.3219

**Table 5:** Practical computational cost of the proposed method.

Dataset	*N*	*d*	Optimizer Iterations	*k*-NN Time (s)	Optimization Time (s)	Total Time (s)
Covtype	5810	10	41	2.3644±0.0006	0.0614±0.0296	2.4332±0.4150
Synthetic 1	1300	2	38	0.0198±0.0085	0.1440±0.0845	0.1669±0.0918
Synthetic 2	5000	2	27	0.0531±0.0392	0.0864±0.0754	0.1520±0.1188

**Table 6:** Comparison of empirical expectation for cohesion metrics.

Dataset	Algorithm	IV	CMD	DMD
Covtype	Physics-Based	173,473.20 ± 65,737.32	9.45 ± 2.35	3.16 ± 0.61
	Kneedle	12.82 ± 2.74	1.01 ± 0.03	0.58 ± 0.01
Synthetic 1	Physics-Based	212.09 ± 184.39	1.59 ± 0.38	0.52 ± 0.11
	Kneedle	0.01 ± 0.00	0.05 ± 0.01	0.03 ± 0.00
Synthetic 2	Physics-Based	8248.37 ± 1734.76	6.52 ± 1.21	1.56 ± 0.25
	Kneedle	0.08 ± 0.03	0.12 ± 0.01	0.05 ± 0.00

**Table 7:** Comparison of empirical expectation for inter-cluster separation metrics.

Dataset	Algorithm	Silhouette	Davies–Bouldin	Calinski–H.
Covtype	Physics-Based	0.4202 ± 0.0187	1.5979 ± 0.1874	77.22 ± 17.15
	Kneedle	−0.5150 ± 0.0089	1.4868 ± 0.0282	11.12 ± 0.90
Synthetic 1	Physics-Based	0.3132 ± 0.2325	2.4542 ± 0.8381	315.61 ± 246.87
	Kneedle	−0.2016 ± 0.0127	1.1583 ± 0.0336	8.40 ± 1.25
Synthetic 2	Physics-Based	0.4588 ± 0.0281	11.2124 ± 10.7219	28.46 ± 6.71
	Kneedle	−0.4658 ± 0.0114	3.8178 ± 1.3769	2.41 ± 0.18

## Data Availability

The Covtype dataset analyzed in this study is publicly available as described in [29]. Synthetic datasets were generated using the scripts provided in the project repository, with a global seed of 23 (see Section 3.1). No new human-subject data were collected.

## Abbreviations

The following abbreviations are used in this manuscript:

ACE	Average Causal Effect
L-BFGS-B:	Limited-memory Broyden–Fletcher–Goldfarb–Shanno (Bound-constrained)
CMD	Cluster Mean Diameter
DAG	Directed Acyclic Graph
DBSCAN	Density-Based Spatial Clustering of Applications with Noise
DMD	Cluster Mean Distance
IV	Intracluster Variance
k-NN	k-Nearest Neighbors
PC	Peter–Clark algorithm
RK4	Fourth-order Runge–Kutta
SCM	Structural Causal Model
SG	Savitzky–Golay
